# Chirurgische Behandlung von Kindern mit Lebertumoren in Deutschland: Evidenz, Versorgungsstruktur und Perspektiven

**DOI:** 10.1007/s00104-025-02438-1

**Published:** 2026-01-07

**Authors:** Juri Fuchs, Christoph W. Michalski, Patrick Günther

**Affiliations:** 1 Sektion Kinderchirurgie, Klinik für Allgemein‑, Viszeral- und Transplantationschirurgie, Universitätsklinikum Heidelberg, Im Neuenheimer Feld 430, 69120 Heidelberg, Deutschland; 2 Service de Chirurgie Pédiatrique et de Transplantation Hépatique Pédiatrique, Hôpital Paris-Bicêtre, APHP, Université Paris-Saclay, Paris, Frankreich; 3 Klinik für Allgemein‑, Viszeral- und Transplantationschirurgie, Universitätsklinikum Heidelberg, Heidelberg, Deutschland

**Keywords:** Pädiatrische Lebertumoren, Hepatoblastom, Leberresektion, Lebertransplantation, Zentralisierung, Pediatric liver tumors, Hepatoblastoma, Hepatectomy, Liver transplantation, Centralization

## Abstract

**Hintergrund:**

Lebertumoren im Kindesalter sind selten und mit hohen therapeutischen Anforderungen verbunden. In den letzten Jahrzehnten konnten durch effektivere Chemotherapie und verbesserte Operationstechniken sowie durch internationale Kooperationen erhebliche Fortschritte erzielt werden. Die Herausforderungen für die chirurgische Therapie bleiben hoch. In Deutschland ergeben sich diesbezüglich spezifische Schwierigkeiten.

**Ziel der Arbeit:**

Zusammenfassung der aktuellen Evidenz zur chirurgischen Behandlung pädiatrischer Lebertumoren, Analyse der Versorgungsstrukturen in Deutschland und Entwicklung von Perspektiven zur Optimierung der Therapie.

**Material und Methoden:**

Narrative Übersicht aktueller Evidenz, systematische Analyse der chirurgischen Ergebnisse vorangegangener Hepatoblastomstudien, Auswertung deutscher Versorgungspfade und dadurch Identifikation von Problemen und Perspektiven.

**Ergebnisse:**

Maßgeblich für die verbesserte Prognose von Kindern mit Lebertumoren (v. a. Hepatoblastom) sind multimodale, risikoadaptierte Therapiekonzepte und verbesserte Operationsstrategien. Aufgrund der Seltenheit von Leberresektionen im Kindesalter befindet sich die pädiatrische Lebertumorchirurgie in Deutschland an einer Schnittstelle zwischen Kinder‑, Viszeral- und Transplantationschirurgie. Chirurgisch entscheidend sind kindgerechte Operationsstrategien zur Erhöhung der Resektionsrate und Vermeidung postoperativer Komplikationen. Eine direkte Übertragung von Erkenntnissen aus der Leberchirurgie bei Erwachsenen birgt hohe Risiken. In Deutschland bestehen gute Grundvoraussetzungen, die Versorgung kann jedoch durch verbesserte Kommunikation mit Referenzstrukturen, intelligente Zentralisierungsansätze und Investition in die chirurgische Ausbildung verbessert werden.

**Diskussion:**

Die Lebertumorchirurgie bei Kindern ist aufgrund ihrer Seltenheit und des hohen Anspruchs mit spezifischen Herausforderungen verbunden. In Deutschland kann die Stärkung multidisziplinärer Strukturen und Kommunikation sowie eine effiziente Zentralisierung der Therapieplanung die Patientensicherheit und Behandlungsergebnisse für Kinder mit Lebertumoren weiter optimieren.

## Abstract (en)

**Background:**

Liver tumors in childhood are rare and associated with high treatment demands. Over the past decades substantial progress has been achieved through effective chemotherapies and improved surgical techniques as well as international collaborations. Nevertheless, the challenges for the surgical treatment remain high. In Germany additional specific difficulties arise in this context.

**Objective:**

To summarize the current evidence on the surgical management of pediatric liver tumors, to analyze the care structures in Germany and to develop perspectives for optimizing treatment.

**Material and methods:**

Narrative review of the current evidence, systematic analysis of the surgical results of previous hepatoblastoma studies, and evaluation of German care pathways in order to identify problems and perspectives.

**Results:**

The improved prognosis of children with liver tumors (particularly hepatoblastoma) is mainly due to multimodal, risk-adapted treatment concepts and advances in surgical strategies. Because of the rarity of pediatric liver resections, pediatric liver tumor surgery in Germany is positioned at the intersection of pediatric, visceral and transplantation surgery. Surgically, strategies adapted to children are crucial to increase resection rates and avoid postoperative complications. A direct transfer of concepts from adult liver surgery carries substantial risks. In Germany the basic prerequisites are good but care can be further improved through better communication with reference structures, intelligent centralization approaches and investment in surgical training.

**Discussion:**

Pediatric liver tumor surgery is associated with specific challenges due to the rarity and high demands. In Germany, strengthening multidisciplinary structures and communication as well as efficient centralization of treatment planning, can further improve patient safety and outcomes for children with liver tumors.

Die Behandlung von Kindern mit Lebertumoren stellt ein hoch spezialisiertes Feld der Kindermedizin dar [2, 23, 27, 44, 47]. Dabei ist die chirurgische Tumorresektion in fast allen Fällen ein zentraler Therapiebestandteil [2, 23, 27, 29, 36, 44, 47]. Aufgrund der Seltenheit dieser Erkrankungen, der komplexen Anatomie und Physiologie des hepatobiliären Systems, dessen zentraler metabolischer Rolle und potenziell lebensbedrohlicher Komplikationen wie Blutungen und Luftembolien, ist die Kinderleberchirurgie sowohl aus Sicht der Kinderchirurgie als auch aus Sicht des gesamten Behandlungsteams außerordentlich anspruchsvoll [2, 27, 44, 50]. Die Lebertumorchirurgie ist – auch im Vergleich zu anderen hepatobiliären Operationen im Kindesalter wie der Kasai-Portoenterostomie bei Gallengangsatresie – mit spezifischen Herausforderungen und Risiken verbunden [2, 8, 12, 23].

Die Behandlung pädiatrischer Lebertumoren wie dem Hepatoblastom, dem häufigsten bösartigen Lebertumor des Kindesalters, verlangt detaillierte Kenntnisse verschiedenster Therapiestrategien, eine hochfunktionale interdisziplinäre Kommunikation und erfordert oft komplexe Operationen [2, 8, 27, 29, 44, 47]. Zur Beurteilung der Resektabilität und für die Operationsplanung sind zum einen profunde Erfahrungen in der Leberchirurgie, zum anderen aber auch spezifische Kenntnisse der Tumorbiologie (z. B. Ansprechen auf Chemotherapie) der pädiatrischen Lebertumoren sowie der kindlichen Physiologie unentbehrlich [11, 12, 44, 47]. Anders als z. B. die Kasai-Portoenterostomie, ist insbesondere die Leberresektion ein Eingriff, der nur von sehr wenigen Kinderchirurgen und -chirurginnen erlernt und regelmäßig durchgeführt wird. Nicht zuletzt deshalb befindet sich die chirurgische Behandlung dieser kleinen Patienten meist in einer Grauzone zwischen Kinderchirurgie, Viszeral- und Transplantationschirurgie der Erwachsenen [12, 33, 41]. Dass mit dieser Schnittstellenproblematik spezifische Herausforderungen und Risiken einhergehen, soll im Folgenden beleuchtet werden.

Auch infrastrukturell und organisatorisch besteht in Deutschland Verbesserungspotenzial. So wurden im kürzlich beendeten PHITT (Pediatric Hepatic International Tumour Trial), an dem die meisten europäischen Länder, die USA und Japan teilnahmen, aus Deutschland in Relation zu seiner Gesamtbevölkerung eine zu geringe Anzahl an Patienten mit Hepatoblastom und hepatozellulärem Karzinom (HCC) eingeschlossen (Präsentationen der Zahlen im Rahmen von SIOPEL (Childhood Liver Tumors Strategy Group Europe) Meetings 2023 und 2024 in Gdansk und Cardiff). Dies deutet darauf hin, dass einige Kinder offenbar nicht in laufende internationale Studien eingeschlossen werden und womöglich ein Informationsmangel besteht.

Auch aus wissenschaftlicher Sicht ist eine Lücke sichtbar: Während für Erwachsene Leitlinien und evidenzbasierte Algorithmen in der Lebertumorchirurgie etabliert sind [22, 25], fehlt es in der pädiatrischen Leberchirurgie an standardisierten und evidenzbasierten Guidelines [12, 27, 30]. Entscheidungen beruhen oft auf extrapolierten Daten aus der Erwachsenenmedizin, obwohl die Pathophysiologie, Tumorbiologie und postoperative Regeneration im Kindesalter grundlegend anders sind [11, 23]. Ziel dieses Artikels ist es, den aktuellen Stand in der chirurgischen Behandlung von Kindern mit Lebertumoren darzulegen, zentrale Herausforderungen in Deutschland herauszustellen und Perspektiven für Forschung, klinische Praxis, Zentralisierung und Ausbildung in der Kinderleberchirurgie aufzuzeigen.

## Epidemiologie: pädiatrische Lebertumoren in Deutschland

Lebertumoren im Kindesalter sind insgesamt selten. Hepatoblastome treten mit einer Inzidenz von etwa 2 bis 2,5 Fällen pro Jahr pro 1 Mio. Kinder auf und stellen die häufigste Indikation für Leberresektionen im Kindesalter dar [18, 39]. Dies entspricht etwa 25 bis 30 Neuerkrankungen pro Jahr in Deutschland [40]. Andere pädiatrische Lebertumoren mit Operationsindikation sind noch seltener, darunter das undifferenzierte embryonale Lebersarkom, hepatozelluläre Karzinome (HCC), hier insbesondere das fibrolamelläre HCC bei jugendlichen Mädchen, Rhabdoidtumoren der Leber, biliäre Rhabdomyosarkome, mesenchymale Hamartome oder auch Lebermetastasen bzw. direkte Invasion per continuitatem durch Nephroblastome oder andere solide Neoplasien [2, 27, 45, 47]. Selten rechtfertigen auch benigne Läsionen wie die fokal noduläre Hyperplasie (FNH), Leberadenome oder Hämangiome eine Leberresektion [16]. Auf Basis der verfügbaren Daten kann extrapoliert werden, dass bei ca. 60 bis 70 Kindern (< 18 Jahre) pro Jahr in Deutschland eine Leberteilresektion bei Lebertumor notwendig ist.

## Leberresektion bei pädiatrischen Lebertumoren: aktueller Stand der Evidenz

Die Leberresektion ist ein zentraler Pfeiler in der Behandlung pädiatrischer Lebertumoren [2, 27, 28, 30, 45, 47]. Die einzigartige Fähigkeit der Leber zu regenerieren, wieder auf ihre ursprüngliche Größe anzuwachsen und die volle Funktion zurückzuerlangen – selbst nach Entfernung von mehr als zwei Dritteln ihres Volumens – eröffnet Chirurginnen und Chirurgen ein breites Spektrum an Optionen zur Tumorresektion [11, 12, 23]. In den letzten Jahrzehnten haben Verbesserungen in der onkologischen Therapie, im anästhesiologischen Management, in der Operationstechnik, im anatomischen Verständnis und in der präoperativen Planung die Grenzen der Lebertumorchirurgie bei Kindern kontinuierlich verschoben [23].

### Historische Entwicklung und die GPOH-Studien

**Abb. 1:**
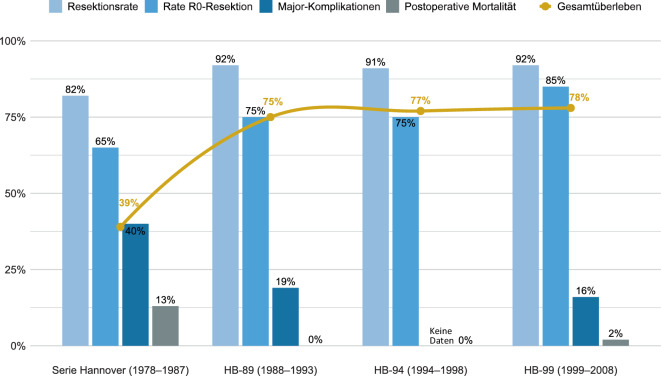
Entwicklung der Ergebnisse der deutschen Hepatoblastomstudien mit Fokus auf chirurgisch relevant Daten

Anders war dies noch in den 1980er-Jahren: Seither hat sich die Therapie pädiatrischer Lebertumoren – insbesondere des Hepatoblastoms – von einer Ära, die durch die Tumorresektion als einzigen Therapieansatz und von hoher Mortalität geprägt war [31] – hin zu multimodalen, risikoadaptierten Therapieregimen mit perioperativer Chemotherapie entwickelt [1, 29, 36, 47]. Diese Fortschritte der 1990er- und frühen 2000er-Jahre sind nicht zuletzt auch dem großen Verdienst der deutschen Hepatoblastomstudien der Gesellschaft für Pädiatrische Onkologie und Hämatologie (GPOH) zu verdanken. Ausgehend von der Hannoveraner Gruppe um Hermann Mildenberger sowie später Dietrich von Schweinitz und Jörg Fuchs wurden mit HB-89, HB-94 und HB-99 3 zentrale deutschlandweite Therapiestudien durchgeführt (Tab. 1; [4, 15, 46]). Diese trugen wesentlich zur dramatischen Verbesserung der Prognose und zur Verfeinerung der chirurgischen Strategien bei Kindern mit Lebertumoren bei (Abb. 1).

**Tab. 1:** Übersicht der Studien zum Hepatoblastom in Deutschland und deren chirurgische Ergebnisse

Studie	Rekrutierungszeitraum	Anzahl Patienten	Resektionsrate	Rate postoperativer Major-Komplikationen	Postoperative Mortalität	Resektionsstatus	Gesamtüberleben
Serie Hannover [31]	1977–1987	28	82 % (23/28) (18 % ohne Resektion [5/28])	40 % (9/23)	13 % (3/23) (90-Tage postoperativ: 48 % [11/23])	R0: 65 % (15/23) R1: 30 % (7/23) Transplantation: 4 % (1/23)	39 % (1-Jahres-Gesamtüberleben)
HB-89 [46]	1988–1993	71	92 % (65/71)	19 % (12/65) Gesamtzahl Major-Komplikationen: 20	0	R0: 75 % (49/65) R1: 22 % (14/65) R2: 2 % (1/65) Transplantation: 2 % (1/65)	75 % (3-Jahres-Gesamtüberleben)
HB-94 [15]	1994–1998	69	91 % (63/69)	Keine detaillierten Angaben	0	R0: 75 % (47/63) R1/R2: 22 % (14/63) Transplantation: 3 % (2/63)	77 % (5-Jahres-Gesamtüberleben)
HB-99 [4]	1999–2008	142 (126 für Auswertung von Komplikationen verfügbar)	92 % (131/142)	16 % (20/126)	2 % (2/126)	R0: 85 % (112/131) R1: 8 % (11/131) R2: 2 % (3/131) Transplantation: 4 % (5/131)	78 % SR: 94 % HR: 63 % (5-Jahres-Gesamtüberleben)

Major-Komplikation definiert als Komplikation, die eine Re-Intervention/Re-Operation erfordert und/oder ein Organversagen verursacht

*SR* Standardrisikogruppe: PRETEXT I, II und III, ohne extrahepatische Tumormanifestation

*HR* Hochrisikogruppe: PRETEXT IV oder Nachweis einer extrahepatischen Erkrankung (Metastasen oder extrahepatische abdominelle Erkrankung oder Beteiligung der Pfortader/Lebervenen)

### SIOPEL-Kooperation und Standardisierung durch PRETEXT (Pretreatment Extent of Tumor)

**Abb. 2:**
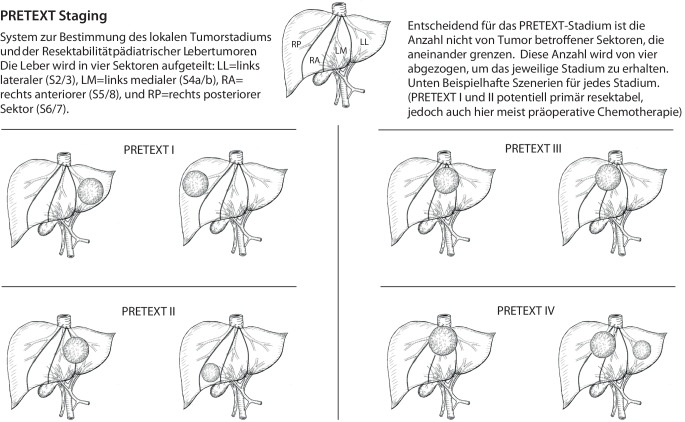
PRETEXT-Staging

Ab den 2000er-Jahren beteiligte sich Deutschland an den europaweiten SIOPEL-Studien, die mit der Einführung des PRETEXT-Stagings (Abb. 2) und einer risikoadaptierten Therapie weitere entscheidende Fortschritte ermöglichten (Tab. 2; [1, 7, 34, 35, 38, 51, 52]). Unter anderem durch die Einführung Cisplatin-haltiger Regime, der konsequenten Anwendung des sog. PRETEXT-Stagings zur Resektabilitätsbeurteilung und Risikostratifizierung (Abb. 2; [1, 15, 29, 44]) und Etablierung interdisziplinärer Teams [48] stiegen die Überlebensraten von etwa 20–35 % in frühen Kohorten auf heute ~75–95 % je nach Risikogruppe ([29]; Tab. 1 und 2 und Abb. 1 und 3). Die Resektion bleibt in fast allen Fällen der zentrale Therapieschritt. Es zeigten sich jedoch auch die Schwierigkeiten bei der Erfassung detaillierter chirurgischer Daten in den Registerstudien (Tab. 1 und 2).

**Tab. 2:** SIOPEL-Studien und deren chirurgische Ergebnisse

Studie	Rekrutierungszeitraum	Anzahl Patienten	Resektionsrate	Rate postoperativer Major-Komplikationen	Postoperative Mortalität	Resektionsstatus/ Operationstyp	Gesamtüberleben
SIOPEL‑1 [38]	1990–1994	150	85 % (128/150)	18 % (23/128)	5 % (5/128)	R0: 80 % (103/128) R1: 12 % (15/128) R2: 3 % (4/128) Transplantation: 5 % (6/128)	75 % (5-Jahres-Gesamtüberleben)
SIOPEL‑2 [35]	1995–1998	135 (77 SR, 58 HR)	84 % (114/135) SR: 97 % (75/77) HR: 67 % (39/58)	Keine detaillierten Angaben	4 % (4/114)	R0: 74 % (84/114) R1: 17 % (19/114) R2: 4 % (4/114) Transplantation: 6 % (7/114)	75 % SR: 91 %, HR: 53 % (3-Jahres-Gesamtüberleben)
SIOPEL‑3 [52]	1998–2006	405 (255 SR, 151 HR)	88 % (357/405) SR: 94 % (242/255) HR: 76 % (115/150)	Keine detaillierten Angaben	2 % (7/357)	R0: 70 % (281/357) R1: 11 % (39/357) R2: 2 % (6/357) Transplantation: 9 % (31/357)	85 % SR: 95 % HR: 69 % (3-Jahres-Gesamtüberleben)
SIOPEL‑4 (nur HR-Patienten) [51]	2005–2009	62 HR	92 % (57/62)	Keine detaillierten Angaben	4 % (2/57)	R0: 56 % (32) R1: 9 % (5/57) R2: 7 % (4/57) Transplantation: 28 % (16/57)	HR: 83 % (3-Jahres-Gesamtüberleben)
SIOPEL‑6 (nur SR-Patienten) [7]	2007–2014	109 SR	100 % (109/109)	Keine detaillierten Angaben	Keine detaillierten Angaben	Keine detaillierten Angaben Transplantation : 7 % (8/109)	SR: 95 % (3-Jahres-Gesamtüberleben)

Major-Komplikation definiert als Komplikation, die eine Re-Intervention/Re-Operation erfordert und/oder ein Organversagen verursacht

*SR* Standardrisikogruppe: PRETEXT I, II und III, ohne extrahepatische Tumormanifestation

*HR* Hochrisikogruppe: PRETEXT IV oder Nachweis einer extrahepatischen Erkrankung (Metastasen oder extrahepatische abdominelle Erkrankung oder Beteiligung der Pfortader/Lebervenen)

**Abb. 3:**
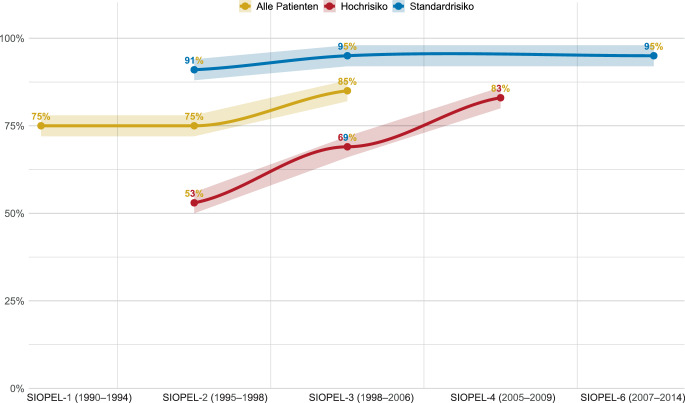
Entwicklung des Gesamtüberlebens in den SIOPEL-Studien

**Abb. 4:**
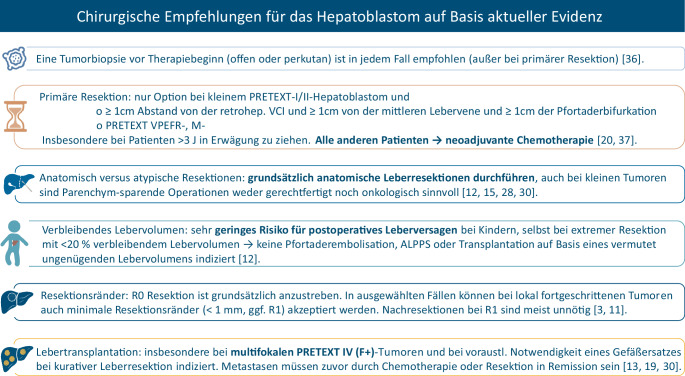
Kernempfehlungen für die chirurgische Behandlung des Hepatoblastoms

Die umfassende globale Datensynthese der Children’s Hepatic Tumors International Collaboration (CHIC; 1605 Patienten mit Hepatoblastom, Therapieepochen 1988–2008) führte zu einem international konsentierten Risikostratifikationsschema [5, 20, 29], das heute die großen Studiengruppen (SIOPEL/GPOH/Children’s Oncology Group (COG)/Japanese Study Group for Pediatric Liver Tumor (JPLT)) und das kürzlich beendete PHITT-Protokoll strukturiert [32, 37]. Die aktuell international konsentierten bzw. auf Basis aktueller Evidenz gültigen wesentlichen Empfehlungen für die chirurgische Behandlung des Hepatoblastoms sind in der Abb. 4 zusammengefasst.

Trotzdem bleiben bislang Details zu Operationen in internationalen Auswertungen oft unterrepräsentiert, sodass es an belastbarer Evidenz zu spezifischen chirurgischen Fragestellungen und Ansätzen zur Verbesserung der Operationen mangelt [9, 12, 17, 28, 30]. Dies umfasst unter anderem die Rolle atypischer versus anatomischer Leberresektionen, Auswirkungen tumorpositiver Absetzungsränder, kindgerechte Schwellen für das verbleibende Restlebervolumen, Definition und Prädiktion des postoperativen Leberversagens, Transplantation versus komplexe Resektionen mit Gefäßrekonstruktion sowie die standardisierte Erfassung von kinderspezifischen Komplikationen in der Leberchirurgie [9, 12, 13, 17]. Diese offenen Fragen werden in der aktuellen Literatur ausdrücklich adressiert und bilden einen Kern laufender und zukünftiger chirurgischer Studien [11–13, 19, 23, 27, 36].

### Besonderheiten der Lebertumorchirurgie im Kindesalter

**Abb. 5:**
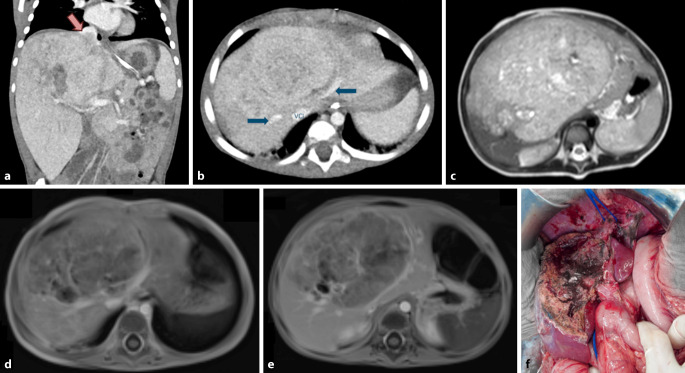
**a**–**f** Fall eines 25 Monate alten Mädchens mit lokal ausgedehntem, multifokalem und synchron pulmonal metastasiertem Hepatoblastom, das bei Diagnose alle Lebersektoren betraf (1C). Außerdem bestand ein Tumorthrombus in der suprahepatischen V. cava (**a**), enger Tumorkontakt zur linken und zur rechten Lebervene (4B, mittlere Lebervene thrombotisch verschlossen) sowie ein thrombotischer Verschluss des linken Pfortaderasts (PRETEXT IV V+, F+, M+ und P−, E−, R−, N−, C−). Initial wurde einzig die Lebertransplantation als kurativer Therapieansatz für möglich gehalten. Diese wäre jedoch nur im Falle eines Ansprechens der Lungenmetastasen mit anschließender vollständiger Sanierung vor der Transplantation infrage gekommen. Nach zunächst unzureichendem Ansprechen auf Cisplatin/Doxorubicin wurde vonseiten der Onkologie bereits eine Palliativtherapie angedacht. Erst nach Diskussion mit dem in der Hepatoblastomchirurgie erfahrenen chirurgischen Team wurde auch eine ausgedehnte Leberresektion als Therapieoption erwogen. Schließlich wurde nach geringem Ansprechen des Primärtumors (**d**, **e**) auf eine Zweitlinientherapie eine Trisektorektomie links (mit atypischer Erweiterung im Segment 7 hinter der rechten Lebervene) sowie mit Cavotomie und Thrombektomie durchgeführt (**f**); histopathologisch wurde eine R0 Resektion bestätigt. Es verblieb der Großteil des rechts posterioren Sektors (Segment 6/7). Die Patientin erholte sich von der Operation ohne Komplikationen. Nach insgesamt 4 Thorakotomien mit Metastasektomien (2-mal links, 2‑mal rechts) ist die Patienten aktuell ohne Chemotherapie tumorfrei und in gutem Allgemeinzustand

Aufgrund der Seltenheit von Lebertumoren im Kindesalter und der unzureichenden Zentralisierung in der Kinderchirurgie, führen die meisten Kliniken nur sehr wenige Leberresektionen bei Kindern durch, auch deshalb mangelt es an Studien und Evidenz [9]. Daher werden oft Strategien aus der Erwachsenenleberchirurgie direkt auf Kinder übertragen [10]. Dieses Vorgehen birgt jedoch erhebliche Risiken, da die besondere Physiologie von Kindern und die Spezifika der pädiatrischen Leberchirurgie dabei meist nicht ausreichend berücksichtigt werden [12] (s. Fallbeispiel, Abb. 5). Wir haben z. B. in der bislang größten Serie pädiatrischer Major-Leberresektionen (*n* = 125) gezeigt, dass bei Kindern nicht die gleichen Grenzen für das minimal notwendige Restlebervolumen gelten wie bei Erwachsenen [12]. Selbst bei extremen Resektionen mit weniger als 20 % verbleibendem Lebervolumen, kam es in keinem einzigen Fall zu einem klinisch relevanten Leberversagen. Bei Erwachsenen ist das postoperative Leberversagen deutlich häufiger und die Haupttodesursache nach Leberresektion; hier gilt eine Grenze von mindestens 25 % als gesetzt [43]. Es ergibt sich eine unmittelbare klinische Relevanz für Therapieentscheidungen, da beispielweise die Entscheidung für eine Transplantation statt einer Leberresektion bei Kindern mit Hepatoblastom mitunter aufgrund eines vermutet unzureichenden Restlebervolumens getroffen wurde [21, 42].

### Postoperative Komplikationen und Einfluss auf das Überleben

Ein weiterer wichtiger Aspekt ist die Entwicklung standardisierter Kriterien zur Bewertung leberspezifischer Komplikationen. Nur bei einheitlicher Erfassung und Dokumentation von unerwünschten Ereignissen werden eine objektive Beurteilung und ein Vergleich von Ergebnissen über verschiedene Zentren hinweg ermöglicht [26]. Diese Standards sind entscheidend, um die Qualität der Versorgung kontinuierlich zu verbessern [9, 26]. Vereinzelte weitere Studien zur Leberresektion im Kindesalter konnten Risikofaktoren und Unterschiede zur Erwachsenenchirurgie identifizieren [14, 24, 50], viele Fragen sind jedoch noch unbeantwortet [3, 17, 27, 36, 44]. Dass die Senkung der perioperativen Komplikationsrate in der Leberresektion bei Kindern mit Lebertumor von äußerster Wichtigkeit ist, und zwar über die unmittelbare vitale Bedrohung hinaus, wird dadurch verdeutlicht, dass intra- und postoperative Komplikationen signifikant mit schlechterem Gesamtüberleben assoziiert sind [4, 47, 50]. Dies lässt sich unter anderem durch den verzögerten Beginn der postoperativen Chemotherapie bei schweren Komplikationen erklären [47]. Insgesamt unterstreicht dies, dass sowohl weitere chirurgische Forschung als auch die Optimierung bestehender Strukturen in der klinischen Praxis Potenzial zur Verbesserung der Prognose von Kindern mit Lebertumoren haben.

## Versorgungsstruktur in Deutschland

In Deutschland leistet das GPOH-Lebertumorregister seit seiner Einführung im Jahr 2011 einen zentralen Beitrag zur Erfassung und Therapie primärer Lebertumoren im Kindes- und Jugendalter (0 bis 20 Jahre) [47]. Ziele sind u. a. eine umfassende Epidemiologie- und Versorgungsforschung, die Dokumentation der Behandlung, Therapieempfehlungen sowie die Asservierung von Material; pro Jahr werden etwa 35 bis 40 Patient:innen erwartet. Das Register ist zudem international vernetzt (z. B. CHIC) und bildet Empfehlungen im Anschluss an das PHITT-Protokoll ab, das zum 31.12.2023 endete. Diese Strukturen sind fachlich wie organisatorisch eine wichtige Leistung und verdienen große Anerkennung.

### Herausforderungen der Versorgungsstruktur in Deutschland

Gleichzeitig zeigen sich – v. a. aus chirurgischer Perspektive – Grenzen der derzeitigen Struktur: Erstens werden zwar Operationen erfasst, in den bisher zugänglichen Auswertungen stehen jedoch epidemiologische und onkologische Parameter im Vordergrund, und es mangelt an fein granulierten chirurgischen Daten. Zweitens werden bestimmte pädiatrische Lebertumoren in anderen GPOH-Registern (z. B. CWS, EU-RHAB, MAKEI) und nicht parallel im Lebertumorregister erfasst. Drittens erfolgt im klinischen Alltag die Erstmeldung und die weitere Kommunikation zwischen dem lokal behandelnden Zentrum und der GPOH-Studienzentrale häufig über die pädiatrische Onkologie; aus chirurgischer Sicht wird berichtet, dass direkte Fallbesprechungen zwischen Operateuren und Referenzstrukturen nicht regelhaft etabliert sind und ein bidirektionaler, direkter Austausch zwischen Referenzchirurgen und lokal behandelndem chirurgischem Team meist ausbleibt. Da jedoch die Indikationsstellung und die Festlegung der chirurgischen Strategie bei pädiatrischen Lebertumoren spezifische Kenntnisse der Erkrankungen, der kindlichen Physiologie und geeigneter Techniken erfordert, die nur an wenigen Zentren oder bei Einzelpersonen vorhanden sind, kann dieser Kommunikationsmangel zu Problemen führen.

### Struktur der onkologischen Zentren und Spezialisierung in Deutschland

Wie bereits in der Einleitung erwähnt, fällt im internationalen Vergleich auf, dass Deutschland – trotz eigentlich hoher Versorgungsdichte – in der Patientenrekrutierung für das PHITT-Protokoll unterrepräsentiert war. Über die Hintergründe dieser Beobachtung kann letztlich nur spekuliert werden. Eine Ursache könnte in der Verteilung der wenigen Patient:innen auf viele Zentren liegen, da dies eine Standardisierung und effiziente Gestaltung vieler Abläufe erschwert. Derzeit werden in Deutschland 60 Institutionen als GPOH Kooperationskliniken gelistet, und 37 Kliniken sind von der Deutschen Krebsgesellschaft (DKG) als kinderonkologische Zentren zertifiziert. Dass die Zentralisierung der pädiatrischen Onkologie – insbesondere der onkologischen Kinderchirurgie – zahlreiche Prozesse effizienter gestalten kann, konnte z. B. in den Niederlanden gezeigt werden. Dort werden seit 2015 alle Kinder mit soliden Tumoren an einem einzigen Zentrum operiert [49]. Wohl auch deshalb werden komplexe pädiatrische Leberresektionen, z. B. beim Hepatoblastom, in Deutschland mitunter von Leberchirurgen, die sonst nur Erwachsene operieren, durchgeführt [33, 41]. Während hier die technische Expertise und Routine in der hepatobiliären Chirurgie meist groß ist, sind sie nicht Bestandteil des eigentlichen Behandlungsteams und kennen unter Umständen die oben genannten Besonderheiten der kindlichen Physiologie sowie der Tumorbiologie nicht (s. Fallbeispiel, Abb. 5). Dies erschwert die Kommunikation von Referenzempfehlungen, die mitunter nicht oder erst nach bereits durchgeführten Eingriffen weitergegeben werden, woraus falsche chirurgische Strategien resultieren können [10, 12]. Dies ist daher nicht als die optimale Lösung für Kinder mit Lebertumoren zu betrachten. Ein Blick in andere Länder zeigt, dass beispielsweise in Frankreich und Großbritannien Kinder mit Lebertumoren an spezialisierten Zentren für Kinderleberchirurgie von Kinderchirurgen mit speziellem Fokus auf die hepatobiliäre Kinderchirurgie operiert werden [6, 12]. Die Ausbildung solch spezialisierter Kinderchirurgen stellt sich in Deutschland jedoch als sehr schwierig dar. Die geringen Fallzahlen an den meisten Kliniken bei großen onkologischen Eingriffen und den besonders seltenen Leberresektionen erschweren eine strukturierte Ausbildung in der Kinderchirurgie enorm. Darüber hinaus gibt es derzeit kein allgemein etabliertes System für gezielte Weiterbildungs- oder Rotationsprogramme zwischen Kliniken oder Disziplinen (z. B. sog. Fellowships). Auf individueller Ebene bleiben längere Auslandsaufenthalte und/oder die doppelte Facharztausbildung in Viszeral- und Kinderchirurgie als Optionen für eine gezielte Ausbildung in der hepatobiliären Kinderchirurgie.

### Spezifische Herausforderungen in Deutschland

(Siehe auch Abb. 6)

- *Geringe Zentralisierung:* Es gibt weder eine verpflichtende noch eine allgemein akzeptierte, freiwillige Konzentration der Behandlung pädiatrischer Lebertumoren an wenigen Zentren. Dadurch werden Kinder mit Lebertumoren verteilt an vielen Kliniken behandelt, woraus sich eine geringe Fallzahl pro Standort ergibt.
- *Schnittstellenproblematik:* In einigen Kliniken übernehmen Erwachsenenchirurgen die Leberresektionen bei Kindern. Dies kann leberchirurgische Expertise sichern, birgt aber Risiken, da Besonderheiten der kindlichen Physiologie und Tumorbiologie nicht immer ausreichend berücksichtigt werden.
- *Mangel an direkter Kommunikation zwischen Referenzstruktur und behandelnden Chirurgen:* Die Falldiskussionen erfolgen häufig indirekt (z. B. primär über die pädiatrische Onkologie), oft ohne direkten fachlichen Austausch zwischen Referenzärzten und behandelndem Team. Dadurch werden spezifische chirurgische Fragestellungen teils verspätet oder nur indirekt adressiert; das begünstigt Informationsverluste und kann suboptimale Entscheidungen zu Folge haben.
- *Unzureichende Ausbildungsstruktur:* unzureichende Möglichkeiten der Aus- und Weiterbildung in der Kinderchirurgie, um Leberresektionen sicher durchführen zu können. Allgemeine Nachwuchsprobleme in der Chirurgie verschärfen diese Situation.

**Abb. 6:**
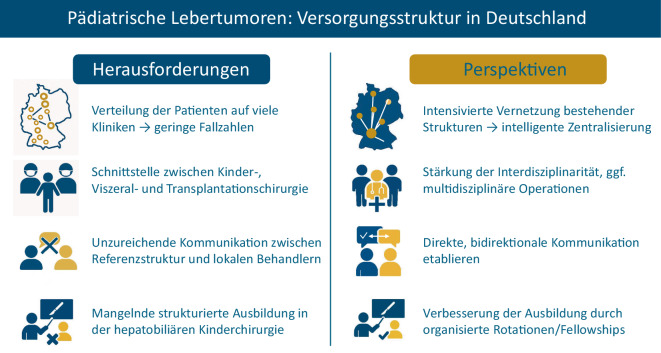
Zusammenfassung der Herausforderungen und möglichen Perspektiven zur Optimierung der Versorgung von Kindern mit Lebertumoren in Deutschland

## Perspektiven für die Kinderleberchirurgie in Deutschland

### Ausbau bestehender Strukturen, Intensivierung der Vernetzung und intelligente Zentralisierung

Die Konzentration der Behandlung aller Kinder mit Lebertumor an 1 bis 2 Zentren in Deutschland, wie in einigen europäischen Ländern etabliert, ist nicht unbedingt notwendig und erstrebenswert. Systematisch verstärkt werden sollte aber die Vernetzung bestehender Strukturen und Kompetenzen [49]. Als Beispiel wären hier zentrale Stellen für die Studienregistrierung der Patienten zu nennen, außerdem transparente Fallkonferenzen mit direkter Kommunikation zwischen Referenz- und operierender Chirurgin/operierendem Chirurgen sowie definierte Rückmeldeschleifen (inklusive chirurgischer Ergebnisberichte) zwischen zentraler Koordinationsstelle und behandelnder Klinik. Die direkte, bidirektionale Kommunikation zwischen Referenzstrukturen und behandelnden Teams könnte als Standard etabliert werden. Es könnten wenige überregionale Referenzzentren für Kinderleberchirurgie als Knotenpunkte fungieren, ohne die wohnortnahe onkologische Betreuung zu schwächen. Über diese Zentren könnte dann auch die aus unserer Sicht so wichtige direkte Kommunikation zwischen behandelndem Team und Referenzzentrum erfolgen. Wünschenswert wäre hier ein standardisierter Ablauf von der Registrierung des Patienten über regelmäßige Fallkonferenzen mit direkter Besprechung des Falls, Kommunikation und Diskussion der Therapieempfehlung sowie der Ergebnisse der Eingriffe zwischen Behandlern und Referenzärzten. Darüber hinaus wäre die Aktualisierung deutscher Leitlinien zur Behandlung kindlicher Lebertumoren auf Basis internationaler Empfehlungen erstrebenswert. Übergeordnetes Ziel sollte sein, dass alle Kinder von derselben Qualität der Diagnostik, Indikationsstellung und chirurgischen Expertise profitieren – unabhängig vom Erstvorstellungsort (Abb. 6).

### Stärkung der Interdisziplinarität

Die Einbindung aller beteiligten Disziplinen – von der Erstdiagnose über das Re-Staging bis zur Operationsplanung – sollte der Standard im Rahmen der multidisziplinären Behandlung von Kindern mit Lebertumoren sein. In ausgewählten Situationen können auch gemeinsame Eingriffe von Kinder- und Leberchirurgen aus der Erwachsenenchirurgie sinnvoll sein; Voraussetzung sollte allerdings dann auch die umfassende Einbindung aller beteiligten Chirurgen und Chirurginnen in die Tumorboards und den gesamten Therapieplan der Patienten sein. Außerdem müssen eine kindgerechte perioperative Infrastruktur sichergestellt sowie die oben genannten Besonderheiten der Kinderleberchirurgie beachtet werden. Auch dies könnte durch die oben beschriebenen Fallkonferenzen mit den Referenzstrukturen sichergestellt werden.

### Verbesserung der Ausbildung durch national koordinierte Maßnahmen

Eine gute chirurgische Versorgung von Kindern mit Lebertumoren kann langfristig nur mit einer gezielten Ausbildung von Kinderchirurgen und -chirurginnen mit Spezialisierung auf die hepatobiliäre Chirurgie sichergestellt werden. Unterstützt durch Fachgesellschaften könnten nationale oder internationale Fellowships und Rotationen in Zentren für geeignete Kandidat:innen eingerichtet werden. Dadurch ließe sich auch eine für Deutschland bedarfsgerechte Spezialisierung steuern, und die Kinderleberchirurgie könnte aus der Grauzone zwischen den Disziplinen heraustreten. Möglicherweise kann zukünftig auch die Weiterentwicklung von Simulationstrainings helfen, um seltene, komplexe Szenarien standardisiert zu erlernen. Klare Weiterbildungspfade erhöhen die Attraktivität und könnten so dem Nachwuchsmangel entgegenwirken.

### Forschungsfelder und zukünftige Entwicklungen in der pädiatrischen Leberresektion

Neben den bereits angesprochenen Aspekten wie der Standardisierung von Ergebnisberichten und Komplikationen, der detaillierteren Erfassung chirurgischer Daten in internationalen Studienprotokollen (z. B. PHITT II), der Besonderheiten der pädiatrischen Leberresektion in Abgrenzung zur Leberchirurgie bei Erwachsenen, können technologische Innovationen diese Entwicklungen flankieren. Mit künstlicher Intelligenz gestützte Bildanalyse mit 3D-Rekonstruktionen könnte die präoperative Planung verbessern [12]. Mittels Resektionssimulationen oder auch prädiktiven Modellen zur Gefäßinfiltration könnte die Resektabilitätsbeurteilung genauer, die Planung der Eingriffe spezifischer, und die Operation dadurch sicherer gemacht werden. Dabei muss stets das Ziel nicht der Technikselbstzweck, sondern die robuste Unterstützung konkreter klinischer Entscheidungen (z. B. Resektion vs. Transplantation, Notwendigkeit von Gefäßrekonstruktionen) und die Reduktion vermeidbarer Komplikationen sein.

## Zusammenfassung

Pädiatrische Lebertumoren sind selten, und ihre Therapie stellt hohe Ansprüche an die Behandler. Durch die Einführung multimodaler, risikoadaptierter Therapien und die Erhöhung der Operationssicherheit haben sich die Ergebnisse in den letzten Jahrzehnten deutlich verbessert.

In Deutschland bestehen gute Grundstrukturen wie das GPOH-Lebertumorregister, zugleich gibt es aber Verbesserungspotenzial: Die Rekrutierung in internationalen Protokollen war zuletzt unzureichend, und die ausbaufähige Kommunikation mit Referenzstrukturen sowie Ausbildungshürden in der Kinderleberchirurgie begünstigen eine heterogene Praxis – bis hin zur Durchführung komplexer Resektionen durch Erwachsenenchirurg:innen außerhalb kinderonkologischer Kernteams. Diese Situation birgt vermeidbare Risiken, da gezeigt wurde, dass zentrale Dogmen der Erwachsenenchirurgie nicht ohne Weiteres auf Kinder übertragbar sind. Die verfügbaren Daten unterstreichen vielmehr die Besonderheiten der kindlichen Leberchirurgie und die Notwendigkeit spezifisch pädiatrischer Expertise.

Konkrete Schritte für die Zukunft umfassen die Erarbeitung standardisierter deutscher Leitlinien für pädiatrische Lebertumoren und den Ausbau einer intelligenten Vernetzung zwischen Referenzstrukturen und behandelndem Zentrum mit direkter Kommunikation. Ergänzend sollten gezielte Weiterbildungswege (Rotationen/Fellowships) für die Kinderleberchirurgie etabliert werden. Weitere spezifische Forschung im Feld der Kinderleberchirurgie kann Evidenzlücken verkleinern. Dadurch kann die Komplikationsrate weiter gesenkt und die Prognose von Kindern mit Lebertumoren in Deutschland weiter verbessert werden.

## Fazit für die Praxis

- Pädiatrische Lebertumoren sind selten und erfordern auf Kinder spezialisierte chirurgische Expertise.
- Multimodale, risikoadaptierte Konzepte basierend auf dem PRETEXT-Staging und verbesserte Operationstechniken haben die Prognose für Kinder mit Hepatoblastom auf > 80 % 5‑Jahres-Überleben verbessert.
- Zentraler Pfeiler für die Heilung bleibt neben der Chemotherapie die komplette Tumorresektion; hier unterscheiden sich kindgerechte Strategien von der Erwachsenenleberchirurgie.
- Postoperative Komplikationen wirken sich direkt auf das Gesamtüberleben aus; deren Reduktion ist ein zentrales Ziel und wird zur weiteren Prognoseverbesserung führen.
- In Deutschland bestehen gute Strukturen (z. B. GPOH-Lebertumorregister), doch fehlende Zentralisierung, Schnittstellenprobleme und Ausbildungshürden führen zu heterogener Versorgung.
- Verbesserte Kommunikation zwischen Referenz- und Behandlungszentren, gezielte Weiterbildung (Fellowships/Rotationen) und nationale Leitlinien können die Versorgung weiter optimieren.
